# Improved Production of *Streptomyces* sp. FA1 Xylanase in a Dual-Plasmid *Pichia pastoris* System

**DOI:** 10.3390/cimb43030161

**Published:** 2021-12-18

**Authors:** Wei Xia, Mengkai Hu, Yang Pan, Dan Wu, Jing Wu

**Affiliations:** 1 State Key Laboratory of Food Science and Technology, Jiangnan University, Wuxi 214122, China; weixia@jiangnan.edu.cn (W.X.); mkaihu@163.com (M.H.); ypan2021@163.com (Y.P.); 2 Key Laboratory of Industrial Biotechnology, School of Biotechnology, Ministry of Education, Jiangnan University, Wuxi 214122, China; 3 International Joint Laboratory on Food Safety, Jiangnan University, Wuxi 214122, China

**Keywords:** *Pichia pastoris*, non-methanol induction, dual plasmid, promoter P*_GCW14_*, xylanase

## Abstract

Methanol is considered as a potential hazard in the methanol-induced yeast expression of food-related enzymes. To increase the production efficiency of recombinant proteins in *Pichia* *pastoris* without methanol induction, a novel dual-plasmid system was constructed, for the first time, by a combining the strategies of genomic integration and episomal expression. To obtain a high copy number of the target gene, the autonomously replicating sequence derived from *Kluyveromyces lactis* (PARS) was used to construct episomal vectors carrying the constitutive promoters P*_GAP_* and P*_GCW14_*. In addition, an integrative vector carrying the P*_GCW14_* promoter was constructed by replacing the P*_GAP_* promoter sequence with a partial P*_GCW14_* promoter. Next, using xylanase XynA from *Streptomyces* sp. FA1 as the model enzyme, recombination strains were transformed with different combinations of integrating and episomal vectors that were constructed to investigate the changes in the protein yield. Results in shake flasks indicated that the highest enzyme yield was achieved when integrated P*_GAP_* and episomal P*_GCW14_* were simultaneously transformed into the host strain. Meanwhile, the copy number of *xynA* increased from 1.14 ± 0.46 to 3.06 ± 0.35. The yield of XynA was successfully increased to 3925 U·mL^−1^ after 102 h of fermentation in a 3.6 L fermenter, which was 16.7-fold and 2.86-fold of the yields that were previously reported for the constitutive expression and methanol-induced expression of the identical protein, respectively. Furthermore, the high-cell-density fermentation period was shortened from 132 h to 102 h compared to that of methanol-induced system. Since the risk of methanol toxicity is removed, this novel expression system would be suitable for the production of proteins related to the food and pharmaceutical industries.

## 1. Introduction

The methylotrophic yeast *Pichia pastoris*, one of the most effective and convenient industrial expression systems [1,2,3,4,5], has been widely used for the production of various heterologous proteins because of its unique advantages, including simple nutritional requirements, growth to a very high cell density, simple purification methods, diverse post-transcriptional modifications, and tight regulation of the induction process [6,7,8]. To date, more than 1000 heterologous proteins have been successfully expressed using this system [9,10,11,12,13,14].

Two types of promoter elements are commonly used in the *P. pastoris* expression system: inducible promoters, represented by the methanol-induced P*_AOX_* promoter [15], and constitutive promoters, represented by the constitutive glyceraldehyde 3-phosphate dehydrogenase P*_GAP_* promoter [16] and the newly discovered constitutive GCW14 cell wall protein P*_GCW14_* promoter [17]. P*_AOX_* is the strongest promoter currently known and can strictly control the expression of heterologous proteins under induction with methanol [18,19,20]. Although many proteins have been successfully expressed using the P*_AOX_* promoter, the use of methanol in large-scale production processes has many drawbacks, including difficulty in measuring the concentration of methanol because of its volatility, the potential fire hazard, and unsuitability for production of food proteins because of its toxicity [6,21]. These serious disadvantages of the P*_AOX_* promoter have prompted research on other promoters. The constitutive *P. pastoris* expression system carrying the P*_GAP_* promoter is the most commonly applied methanol-free system, which does not require methanol for heterologous expression, and could be cultivated in a continuous high-cell-density fermentation mode [16,22]. However, its expression efficiency is much weaker than that of P*_AOX_* [23,24,25,26,27]. Another recently identified constitutive promoter, P*_GCW14_*, has an equally strong transcription initiation intensity and significantly shorter fermentation time compared to that of P*_AOX_* [17,28]. Therefore, expression systems carrying P*_GCW14_* are expected to replace those carrying P*_AOX_* for the large-scale production of heterologous proteins, particularly proteins used in the food and healthcare industries, which need higher safety requirements [29,30,31].

According to the differences in the locations of gene expression cassettes between different types of vector plasmids, heterologous genes generally exist in *P. pastoris* in two forms, integrated and episomal [32]. Integrated expression refers to restructuring heterologous genes into the yeast genome through specific homologous sequences, while heterologous genes in episomal plasmids exist in the cytoplasm of yeast cells and are dissociative from the genome without integration. Integrating heterologous genes for expression into the chromosome of *P. pastoris* was once considered to be more stable than using episomal vectors, but the integration sites have been shown to be adjacent and heterologous genes may be lost by homologous recombination under certain conditions [33]. Either the pPIC9K, pPICZα, and pGAPZα plasmids are usually integrated into the yeast genome alone, or simultaneously in some cases, to control and enhance the expression level in recombinant strains [34,35,36,37,38]. However, integrative expression can also adversely affect the yeast’s own physiological metabolism and integration of high-copy heterologous genes into the genome was found to reduce the growth rate and cell activity of recombinant yeasts. Consequently, the integration of heterologous genes into the yeast genome may also result in genetic instability of the host cells, leading to floating and a decrease of the expression level [39]. In contrast, episomal vectors are replicated independently in host cells, leading to benefits such as a significantly increased copy number of inserted genes and little effect on the host genome [10,40]. The autonomously replicating sequence (ARS) on episomal plasmids is the key factor for the stable maintenance of episomal plasmids in host cells [9,41,42,43,44]. A recently identified autonomously replicating sequence derived from *Kluyveromyces lactis* (PARS) has a stronger replication ability than ARS sequences A76 and C937 from *P. pastoris* itself [45,46]. Although the use of episomal vectors to express heterologous proteins in yeast expression systems has certain advantages, there have been few studies on heterologous protein expression in *P. pastoris* using episomal vectors [26]. A stronger constitutive glycosylphosphatidylinositol (GPI) anchoring protein (GCW14) promoter P*_GCW14_* was found, through which the constitutive expression of *Candida antarctica* lipase B (CALB) [17] and *Thermomyces dupontii* thermo-alkaline lipase [47] were higher than P*_GAP_* and P*_TEF1_* promoters. Liang and colleagues used the constitutive promoter P*_GCW14_* to express green fluorescent protein (EGFP) based on glycerol and methanol as carbon sources. The green fluorescent protein (EGFP) expressed through the P*_GCW14_* promoter is about 10 times more intense than the P*_GAP_* promoter [28]. It can be seen that the P*_GCW14_* promoter has a significant advantage in the expression of heterologous proteins in *Pichia pastoris*.

Xylanase is widely used in diverse industries, including papermaking, pasta production, xylooligosaccharide preparation, fruit and vegetable production, and in feed additives [48,49,50]. In our previous study, a xylanase used in food, XynA from *Streptomyces* sp. FA1 (NCBI accession number: JX560161), was expressed in *P. pastoris* using methanol induction with an enzyme activity of 1374 U·mL^−1^ [51]. In the present study, a strategy combining episomal expression and genomic integrative expression was designed to further improve the expression level of xylanase XynA, and also eliminate the risk of methanol contamination. A new integrative vector carrying the P*_GCW14_* promoter was constructed by partially replacing the P*_GAP_* promoter sequence with the P*_GCW14_* promoter. The remaining fragment of the P*_GAP_* promoter was reserved as the integrating sequence. The autonomously replicating sequence from *K. lactis* (PARS) was inserted into a pGAPZα plasmid backbone for the construction of episomal plasmids carrying the constitutive P*_GAP_* or P*_GCW14_* promoter. Next, different combinations of integrating and episomal plasmids were transformed into the host cell to explore the yield of XynA. This work was aimed to develop a novel dual-plasmid *P. pastoris* expression system to achieve high expression levels without methanol induction, which is of great value for the production of proteins or enzymes with high safety requirements.

## 2. Materials and Methods

### 2.1. Strains and Chemicals

The host strains *E. coli* JM109 and *P. pastoris* KM71 were purchased from Invitrogen (Carlsbad, CA, USA). The expression vectors pMD18-T-simple, pGAPZαA, and pPPIC9K were purchased from Novagen (Shanghai, China). The vector pMD18-T-*xynA* was stored in our laboratory. The restriction enzymes *Not* I, *Eco*R I, *Avr* II, and T4 DNA ligase were purchased from Takara (Dalian, China). Primer synthesis and DNA sequencing were conducted by Shanghai Sangon Biological Engineering Technology & Services Co., Ltd. (Shanghai, China). Beechwood xylan was purchased from Sigma-Aldrich (X4252-25G, St. Louis, MO, USA). The growth mediums YPD, MD, BMGY, BMMY, BSM, G418 (Invitrogen, CA, USA), and zeocin (Sangon Biotech, Shanghai, China) were prepared according to the Multi-Copy *Pichia* Expression Kit (Invitrogen, Carlsbad, CA, USA). Other chemicals were of analytical grade and were purchased from Sinopharm Chemical Reagent Co., Ltd. (SCRC, Shanghai, China). An In-Fusion HD cloning kit was purchased from Takara (639648, Japan).

### 2.2. Construction of the Episomal Vector pGCW14ZαA-PARS

**Figure 1:**
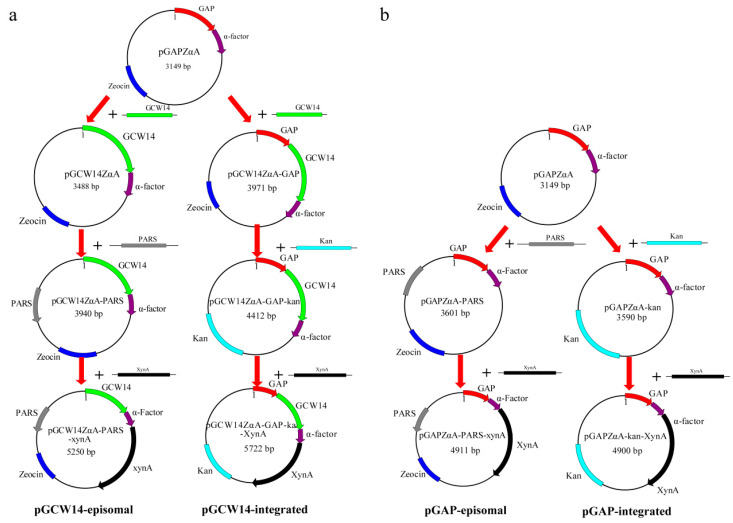
The construction process of episomal or integrative vectors. (**a**) Construction of vectors carrying the P*_GCW14_* promoter; (**b**) Construction of vectors carrying the P*_GAP_* promoter.

The newly constructed episomal vector, pGCW14ZαA-PARS, was designed to be capable of self-replication and amplification outside of the host genome and contained the strong constitutive promoter, P*_GCW14_*. First, the P*_GAP_* promoter in the commercial vector pGAPZαA was substituted with the P*_GCW14_* promoter (see left part of Figure 1a). The P*_GCW14_* promoter (NCBI accession number: XM_002490678) was amplified from the *P. pastoris* genome using the primer set gcw-F1/gcw-R1, and the vector backbone without the P*_GAP_* promoter was amplified from the pGAPZαA plasmid using the primer set gap-F1/gap-R1. The two fragments were purified and ligated using Takara’s In-Fusion HD cloning kit to obtain the promoter substituted vector pGCW14ZαA. Next, the *Pichia* autonomously replicating sequence (PARS) was artificially synthesized and inserted at the position downstream of the pUC origin in the pGCW14ZαA plasmid by the same method described above using primer sets pars-F1/pars-R1 and gcw-F2/gcw-R2 to obtain the episomal plasmid pGCW14ZαA-PARS. All primers used for plasmid construction are shown in Table S1 (See Supplementary Materials).

### 2.3. Construction of the Integrated Vector pGCW14ZαA-GAP-Kan

To construct the integrated vector, pGCW14ZαA-GAP, the P*_GCW14_* promoter was inserted downstream of the P*_GAP_* promoter in the commercial vector pGAPZαA (shown in Figure 1a). The sequence of the P*_GAP_* promoter was employed as the integrated site. For detail, the P*_GCW14_* promoter fragment was amplified from the pGCW14ZαA vector using the primer set gcw-F3/gap-R1, and the vector backbone was amplified from the pGAPZαA commercial vector using the primer set gcw-F1/gap-R2. The two fragments were purified and ligated using Takara’s In-Fusion HD cloning kit to obtain the integrated vector pGCW14ZαA-GAP. Next, the Zeocin resistance gene was substituted for the G418 resistance gene for convenient transformant screening. The vector backbone without the Zeocin resistance gene was amplified from the pGCW14ZαA-GAP vector using the primer set gcw-F4/gcw-R3, and the G418 resistance gene fragment was amplified from the pPIC9K commercial vector using the primer set kan-F/kan-R. The two fragments were also purified and ligated to obtain the integrated vector pGCW14ZαA-GAP-Kan.

### 2.4. Construction of the Episomal Vector pGAPZαA-PARS and the Integrated Vector pGAPZαA-Kan

The episomal vector containing the PGAP promoter, pGAPZαA-PARS, was obtained by inserting the PARS sequence at the position downstream of the pUC origin in the pGAPZαA commercial vector (left part of Figure 1b). Additionally, the integrated vector containing the PGAP promoter, pGAPZαA-Kan, was obtained from the pGAPZαA commercial vector by replacing the Zeocin resistance with G418 resistance using primer sets gcw-F4/gcw-R3 and kan-F/kan-R (right part of Figure 1b).

### 2.5. Construction of Recombinant Vectors for Xylanase Expression

The coding sequence of xylanase XynA was amplified from pMD18-T-*xynA* and inserted into the four vectors described above using the restriction sites *EcoR* I and *Not* I for xylanase expression. The constructed recombinant vectors, pGCW14ZαA-PARS-XynA, pGCW14ZαA-GAP-Kan-XynA, pGAPZαA-PARS-XynA, and pGAPZαA-Kan-XynA were abbreviated as pGCW14-episomal, pGCW14-integrated, pGAP-episomal, and pGAP-integrated, respectively.

### 2.6. Construction of Recombinant Strains

**Figure 2:**
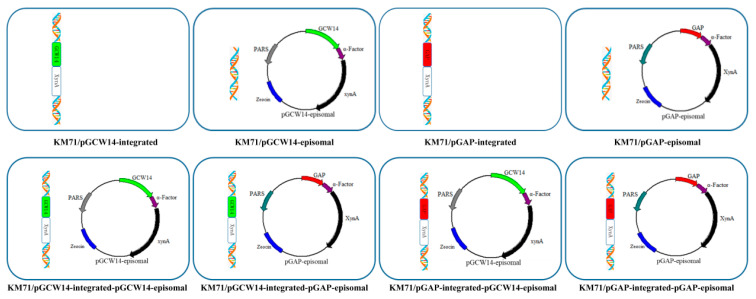
The construction process of the eight recombinant *P. pastoris* KM71 strains with different expression patterns.

Four single-promoter recombinant strains and four dual-plasmid recombinant strains were constructed in this study as shown in Figure 2. The two integrated strains containing a single promoter, KM71/pGCW14-integrated and KM71/pGAP-integrated, were constructed by integrating the expression cassettes of pGCW14-integrated and pGAP-integrated, respectively, into the host genome. First, both of the integrative vectors were linearized by the restriction enzyme *Avr* II and transformed into 80 μL of *P. pastoris* KM71 competent cells by electroporation using a Gene Pulser electroporator (Eppendorf, Hamburg, Germany). Next, 1 mL of pre-cooled 1 M sorbitol solution was added and blown gently. The cells were transferred to a 1.5 mL EP tube and incubated at 30 °C and 200 rpm for 2 h. Transformants were screened on MD plates after incubation for 2–3 days at 30 °C, and further screened using YPD plates containing various concentrations of G418 (1.0–2.0 mg·mL^−1^).

The two episomal strains containing a single promoter, KM71/pGCW14-episomal and KM71/pGAP-episomal, were obtained by directly transforming the vectors pGCW14-episomal and pGAP-episomal, respectively, into *P. pastoris* KM71 competent cells without linearization. Positive transformants were identified using YPDS plates containing 100 μg·mL^−1^ of Zeocin. The four dual-plasmid strains containing a combination of episomal and integrative vectors, KM71/pGCW14-integrated-pGAP-episomal, KM71/pGCW14-integrated-pGCW14-episomal, KM71/pGAP14-integrated-pGCW14-episomal, and KM71/pGAP-integrated-pGAP-episomal, were constructed by transforming the episomal vectors into the competent cells of integrated strains and screening positive transformants on YPDS plates containing 100 μg·mL^−1^ of Zeocin.

### 2.7. Culturing Recombinant Strains in Shake Flasks

Recombinant strains were grown in 10 mL of YPD medium at 30 °C in a shaker (200 rpm) for 24 h. Next, 2.5 mL of culture solution was withdrawn and inoculated into 50 mL of YPD medium containing an additional 4% glycerol. The supernatant was collected after 84 h of culturing to detect the yield of xylanase.

### 2.8. Determination of Genetic Stability of Episomal Vectors

Single colonies of constructed strains containing the *XynA* gene were picked into 2 mL of YPD liquid medium and cultured overnight at 30 °C in a 10 mL shake flask (200 rpm). Next, 1% of the culture was inoculated into fresh YPD liquid medium, and cultured in a 50 mL shake flask at 30 °C and 200 rpm for 2 days. YPD medium was transferred once every two days and sampled, and then the sample was appropriately diluted, coated on a non-selective YPD plate, and cultured at 30 °C. After colony growth, single colonies were randomly picked and seeded on non-selective YPD plates and selective YPD plates (containing 100 μg·mL^−1^ zeocin). The stability of the foreign gene in recombinant strain was determined by comparing the growth numbers of both.

### 2.9. High Density Fermentation in a 3.6 L Bioreactor

The recombinant strain was cultured in a 3.6 L fermenter (Labfors 5, Infors-HT Co., Ltd., Bottmingen, Switzerland) using a constant-rate-fed carbon source mode. The basic medium consisted of 26.7 mL·L^−1^ of 85% phosphoric acid, 0.93 g·L^−1^ CaSO_4_, 18.2 g·L^−1^ K_2_SO_4_, 14.9 g·L^−1^ MgSO_4_·7H_2_O, 4.13 g·L^−1^ KOH, 40.0 g·L^−1^ glycerol, 4.35 mL·L^−1^ PTM1, and histidine 2.0 g·L^−1^. 80% glycerol (histidine 5 g·L^−1^) was added to maintain the carbon source content during the fermentation. The pH of the culture was adjusted to 5.0 using 100% ammonium hydroxide. The dissolved oxygen was controlled at 30% by adjusting the stirring speed and the aeration amount. The dissolved oxygen in the early fermentation is adjusted by the rotation speed. When the maximum rotation speed reaches 800 rpm, pure oxygen is passed into the fermentation to maintain the dissolved oxygen. The supernatant was taken every 12 h to determine enzyme activity, DCW(g·L^−1^), and protein concentration. Glycerol was fed at different speeds (4%, 6%, 10%, 12%) at the carbon-feed stage, and the 10% flow rate was 17.3 g of feed per hour.

### 2.10. Enzyme Activity Assay and Protein Concentration Assay

The substrate was prepared by dissolving 0.5 g of xylan from beechwood in 100 mL of 50 mM phosphate buffer (pH 5.5) and mixing well. The reaction mixture consisted of 1 mL of the above substrate and 1 mL of appropriately diluted enzyme. After incubation at 50 °C for 10 min, 3 mL of 3,5-dinitrosali-cylic acid (DNS) was added to stop the reaction. The reaction solution was boiled for 10 min, rapidly cooled, distilled to a volume of 20 mL with distilled water, and the absorbance was measured at 540 nm (the inactivated enzyme solution that was used as a catalyst was also used as a blank control), and the released xylose equivalent was calculated according to a previously built standard curve. One unit (U) of xylanase activity is defined as the amount of enzyme that releases 1 μmol of reducing sugar per minute from beechwood xylan at 50 °C and pH 5.5 [51]. The protein concentration was determined by the Bradford method with bovine serum albumin (BSA) solutions with gradient concentrations as the standards.

### 2.11. Genomic DNA Extraction and Gene Copy Number Analysis by qPCR

The recombinants were inoculated into 50 mL YPD and cultured at 30 °C for 24 h. The yeast culture was then centrifuged at 10,000 rpm for 15 min to collect the cell pellet. Liquid nitrogen was added to the cell pellet, and then the cell wall was broken by grinding. Next, 150 mg of the above sample was resuspend in 700 μL of pH 8.0 buffer containing 100 mmol·L^−1^ Tris-HCI, 10 mmol·L^−1^ EDTA, and 1% SDS, and incubated at 65 °C for 1 h. The tube was then vortexed briefly and centrifuged at 10,000 rpm for 15 min. The supernatant was extracted twice with 700 μL of chloroform/isoamyl alcohol (24:1). Next, 0.1 mL of 3 M sodium acetate (pH 5.2) solution, 6 μL of RNaseA (10 g·L^−1^), and 1 mL of pre-cooled absolute ethanol were added into the aqueous phase. The liquid was incubated at −20 °C for 30 min to precipitate the DNA completely and then centrifuged at 5000 rpm for 10 min. before the pellet was washed once with 70% ethanol, air-dried, and redissolved in 50 μL TE buffer under 65 °C for 10 min.

GAPDH (accession number XM_002491300) was chosen as the reference gene to detect the copy number of the target gene (*xynA*) in the recombinant strains using the qPCR method. Two sets of qPCR primers were designed (shown in Table S2). The TB Green™ Premix Ex Taq™ II kit (Takara Bio-Inc, Otsu, Japan) and Applied Biosystems StepOnePlus Real-Time PCR System (Thermo Fisher Scientific, Waltham, MA, USA) were used. During the qPCR reaction, the reaction mixture was pre-incubated at 95 °C for 30 s for DNA denaturation and then subjected to 40 cycles, which were 5 min at 95 °C and 30 s at 60 °C. The ΔCt method was used to calculate the copy number of the target gene in *Pichia pastoris*.

## 3. Results

### 3.1. XynA Expressions in Single-Promoter Strains in Shake Flasks

The expression levels of XynA in strains containing different episomal and integrative vectors carrying P*_GAP_* and P*_GCW14_* promoters were compared at a shake flask scale. As shown in Table 1, the expression levels in strains containing episomal vectors were found to be higher than those containing integrative vectors. In particular, the expression level in the strain containing the episomal vector pGCW14-episomal increased 2.82-fold compared to that of the strain containing the integrative vector pGCW14-integrated, demonstrating the advantage of episomal vectors for the expression of heterologous proteins in *P. pastoris*. Further, enzyme activity in the strain containing pGCW14-episomal vectors also increased 2.92-fold compared with that in the strain containing pGAPZαA-episomal vectors, indicating that the initiation efficiency of P*_GCW14_* was much higher than that of P*_GAP_* in the episomal pattern. However, the enzyme activity in the strain containing the pGAPZαA-episomal vector had no significant increase compared with the pGAPZαA-integrated vector, probably due to the weak initiation ability of the P*_GAP_* promoter that could not significantly increase xylanase expression levels, even when used as an episomal vector.

### 3.2. Stability of the pGCW14-Episomal Vector in the Host Cell

It is important to improve the genetic stability of episomal vectors in recombinant strains to avoid adding antibiotics and thus decreasing of expression levels during the whole period (134 h) of fermentation. A previous study reported that the retention rate of PARS vectors after culture for ten generations with and without antibiotics were 97.3% and 39.04%, respectively [46]. However, the method for measurement of vector stability was not clearly presented. In the present study, the pGCW14-episomal vector was constructed using the autonomously replicating sequence PARS and its genetic stability in *P. pastoris* was investigated by repeated experiments. Results indicated that the recombinant *P. pastoris* strain containing pGCW14-episomal had a high genetic retention rate of 84.71% by losing only 15.29% of episomal vectors after the cultured passage for 90 generations (Table 2). *P. pastoris* was generally considered to reproduce 29 generations every 48 h. Therefore, the time for the reproduction of 90 generations was sufficient to support the growth of host cells for more than 144 h, which fully met the growth requirements of industrial, large-scale fermentation of *P. pastoris*. In other words, there was no risk of losing the coding gene of recombinant proteins when using the pGCW14-episomal vector during the whole period of fermentation.

### 3.3. Enhanced XynA Expressions in Dual-Plasmid Strains in Shake Flasks

Novel *P. pastoris* expression systems capable of simultaneous genomic integrative expression and episomal vector expression were constructed by different combinations of two constitutive promoters, P*_GAP_* and P*_GCW14_*, to increase the expression level of XynA. Enzyme activities in the recombinant strains KM71/pGAP-integrated-pGCW14-episomal and KM71/pGCW14-integrated-pGCW14-episomal reached 132.7 ± 9.7 U·mL^−1^ and 112.9 ± 7.2 U·mL^−1^, respectively (data shown in Table 3). These expression levels were more than 2.2-fold higher than those in strains containing the pGAP-episomal vector, indicating that the P*_GCW14_* promoter has a much higher initiation efficiency than the P*_GAP_* promoter in episomal mode, which is in accordance with the results in single-promoter strains. When P*_GAP_* and P*_GCW14_* were integrated separately into the genome for the initiation of expression, expression levels were similar, whereas expression level in the KM71/pGAP-integrated-pGCW14-episomal strain was higher than that in the KM71/pGCW14-integrated-pGCW14-episomal strain. It indicated that competition might occur when the same promoter was used for simultaneous cytoplasmic episomal expression and genomic integrated expression, which is not conducive to the expression of heterologous proteins. However, when the P*_GAP_*-episomal vector was included in the dual-plasmid system, no significant improvement in enzyme activity was observed in the pGCW14-integrated-containing recombinant strain compared with the KM71/pGAP-integrated strain, showing approximately equal yields of about 50 U·mL^−1^ and indicating that the expression pattern of genomic integration might severely limit the initiation efficiency of P*_GCW14_*. The low efficiency of genomic integrative expression could probably be attributed to the complex structure of chromosomes, in which DNA molecules are compressed several thousand times from their primary structure to their quaternary structure [52], resulting in much lower transcription initiation efficiency of heterologous genes in the host genome than in simple episomal vectors.

### 3.4. Gene Copy Number Analysis

**Figure 3:**
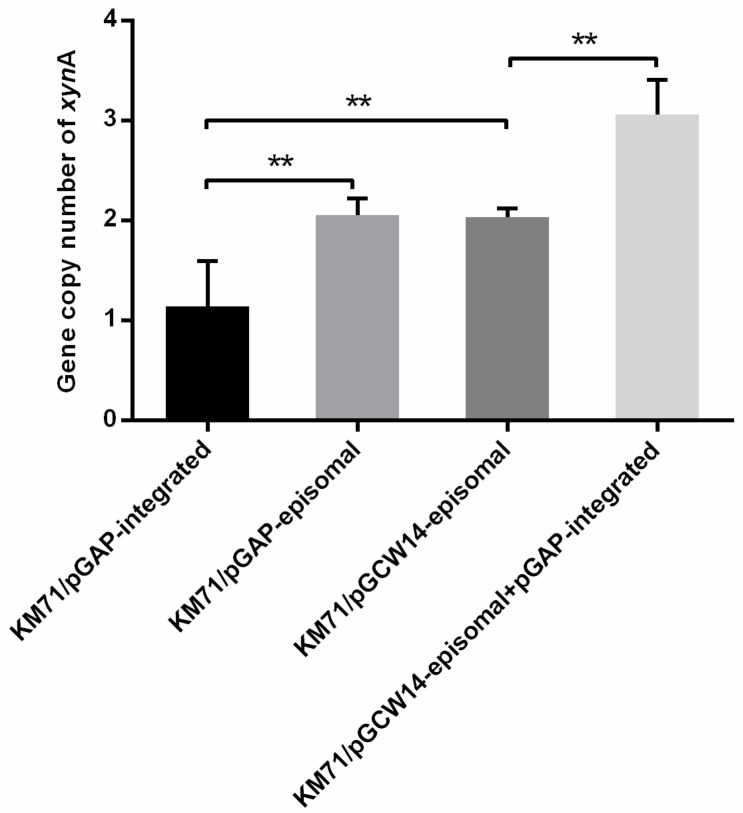
The gene copy number of *xyn*A in four recombinant strains of *P. pastoris* KM71 with different vector combinations. Symbol ** represents that the difference is statistically significant. The significance of difference was analyzed by one-way ANOVA method.

The expression levels of xylanase in different strains were significantly different. In order to better understand the reasons affecting the gene expression level, the representative strains were selected for qPCR analysis in this study. The detailed amplification data are shown in Table S3, and the melt curves and amplification plots are shown in Figure S1. As a result, there were significant differences in the copy number of different recombinant strains. The copy number of the integrated strain KM71/pGAP-integrated was 1.14, the copy number of the episomal strains KM71/pGAP-episomal and KM71/pGCW14-episomal were 2.05 and 2.03, respectively, and the copy number of the new dual-plasmid strain KM71/pGCW14-episomal-pGAP-integrated was 3.06 (Figure 3). The results indicated that the up-regulation of the XynA gene was one of the reasons for the increase in the expression level of the target gene.

### 3.5. Optimization of Feed-Flow Rate of the Carbon Source during Fermentation in the 3.6 L Fermenter

**Figure 4:**
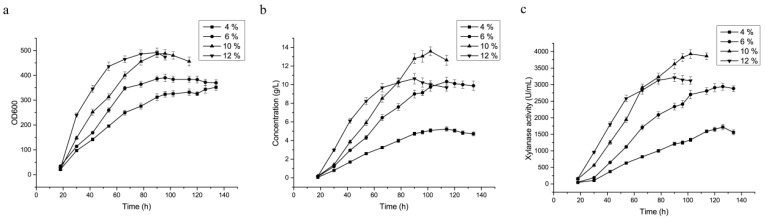
Time course profile of XynA production in the KM71/pGAP-integrated-pGCW14-episomal strain under different carbon-source-feed rates during high-density fermentation in a 3.6 L fermenter. (**a**) Enzyme activity of XynA; (**b**) protein concentration; (**c**) amount of biomass determined by OD_600_ DCW(g·L^−1^). The different carbon-source-feed rates were respectively marked as 4% (■), 6% (●), 10% (▲), and 12% (▼).

Glycerol was chosen as the carbon source for high-cell-density fermentation in a 3.6 L fermenter of the recombinant strain KM71/pGAP-integrated-pGCW14-episomal, which achieved the highest expression level in the shake flask fermentation. Different constant feed-flow rates of glycerol (4%, 6%, 10%, and 12%) were investigated to maximize the expression level of XynA and shorten the fermentation time cycle as much as possible. The main fermentation parameters and comparison of results are shown in Table 4. Results showed that both xylanase activity and protein concentration in the fermentation supernatant reached a maximum when the feed-flow rate of glycerol was 10% (Figure 4a). After 20 h of fermentation at the 12% feed-flow rate, the dissolved oxygen was close to zero. When pure oxygen was supplied to the fermenter and fermentation was carried out for another 20 h, the dissolved oxygen again decreased to zero. After that, the growth and expression efficiency of the yeast was significantly reduced, indicating that when the glycerol supply reached a certain level, fast growth of the yeast depleted dissolved oxygen. This affected further growth of the host cells, thereby inhibiting the expression of the heterologous protein. Enzyme activity under the 10% feed-flow rate reached a maximum at 102 h of cultivation, which was 23.5% shorter than the culture time of 126 h under 4% and 6% feed-flow rates. At the 10% glycerol feeding rate, the enzyme activity, protein concentration, and biomass yield reached 3925 ± 323.8 U·mL^−1^, 13.6 ± 1.1 g·L^−1^_,_ and 8.59 ± 0.69 IU·OD_600_^−1^, respectively, which were 33.5%, 34.6%, and 7.6% higher than the values under the 6% feed-flow rate, respectively (Figure 4). Among them, glycerol was fed at different speeds (4%, 6%, 10%, and 12%) at the carbon feed stage, and the 10% flow rate was 17.3 g of feed per hour.

**Figure 5:**
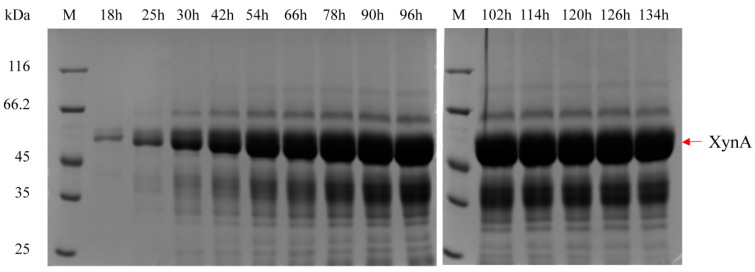
SDS-PAGE analysis of the fermentation supernatant of the recombinant strain KM71/pGAP-integrated-pGCW14-episomal at a 10% glycerol-feeding rate in a 3.6 L fermenter.

The specific activities under four feed-flow rates were similar, indicating that the structure of the recombinant XynA had no improper change and was not affected by the expression rate. In contrast, the rapid expression of the recombinant protein during methanol induction affected the protein structure and caused a significant decrease in specific activities [53]. Further, the highest specific activity of the fermentation broth reached 330.8 U·mg^−1^, which was increased significantly compared to the data of 87 U·mg^−1^ in our previous study [54]. It was possibly due to the larger unit-time productive capacity of XynA reducing the interference of other secreted host proteins. As shown in the SDS-PAGE analysis of the fermentation supernatant of the KM71/pGAP-integrated-pGCW14-episomal strain in the 3.6 L fermenter (Figure 5), a protein of the predicted size (43.0 kDa) for XynA was the main band, which also showed obvious xylanase activity. It could be confirmed that XynA was the primary expression product during the whole period.

### 3.6. Fermentation of Highly Efficient Recombinant Strains in 3.6 L Fermenter

**Figure 6:**
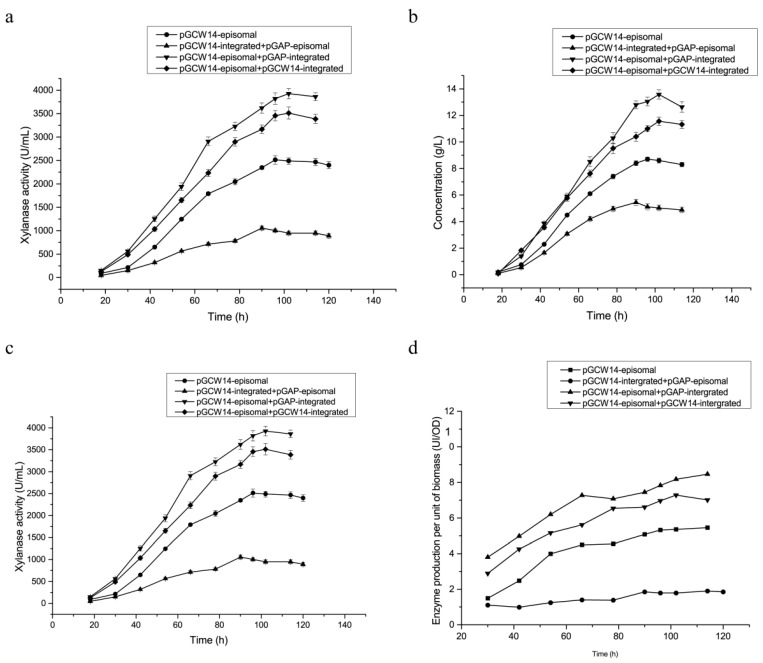
Time-course profile of recombinant xylanase production in constructed *P. pastoris* strains during high-density fermentation in 3.6 L fermenter. (**a**) Enzymes activity of XynA; (**b**) protein concentration; (**c**) amount of biomass determined by OD_600_DCW(g·L^−1^); (**d**) yield per unit of biomass. The carbon-source-feed rate was 10%. The strains were respectively marked as (●) KM71/pGCW14-episomal, (▼) KM71/pGAP-integrated-pGCW14-episomal, (♦) KM71/pGCW14-integrated-pGCW14-episomal, and (▲) KM71/pGCW14-integrated-pGAP-episomal.

The optimized fermentation conditions described above were used to compare the high-cell-density fermentation in a 3.6 L fermenter of the four recombinant strains, which exhibited high expression efficiency in the shake flask, being the KM71/pGAP-integrated-pGCW14-episomal, KM71/pGCW14-integrated-pGCW14-episomal, KM71/pGCW14-integrated-pGAP-episomal, and KM71/pGCW14-episomal strains. The highest enzyme activities and the highest protein yields in the four strains were all achieved at approximately 100 h, which verified that the 10% feed-flow rate of glycerol could indeed shorten the time cycle of high-cell-density fermentation (Figure 6). Furthermore, according to the data shown in Table 5, the total enzyme activities, specific enzyme activities, and amount of protein produced per unit of biomass in the three strains containing the pGCW14-episomal vectors were significantly higher than those in the KM71/pGAP-integrated-pGCW14-episomal strain, indicating that the initiation efficiency of P*_GCW14_* in episomal vectors was still significantly higher than that of P*_GAP_* in the high-density, large-scale fermentation.

Enzyme activity in the KM71/pGCW14-integrated-pGCW14-episomal strain (3512 ± 290.3 U·mL^−1^) was higher than that in the KM71/pGCW14-episomal strain (2512 ± 193.7 U·mL^−1^), but a little lower than that in the KM71/pGAP-integrated-pGCW14-episomal strain. It indicated that the pGCW14-episomal vectors and the pGCW14-integrated vectors have an additive effect, but the additive effect of promoters with different properties, P*_GCW14_* and P*_GAP_*, was the strongest. Enzyme activity in the KM71/pGAP-integrated-pGCW14-episomal strain reached 3925 ± 323.8 U·mL^−1^, which is equal to 16.7-fold of the reported enzyme activity of 235 U·mL^−1^ in a previously constructed recombinant *P. pastoris* strain containing a pGAP-integrated vector [54]. The specific activity was also increased by 2.3-fold. In addition, when compared with the enzyme activity of 1374 U·mL^−1^ and specific activity of 218.1 U·mg^−1^ obtained at 132 h using methanol to induce the expression of the identical *xynA* gene [51], activities achieved in the present work increased by 1.86-fold and 32.1%, respectively.

## 4. Discussion

The methanol-induced *P. pastoris* expression system has been applied in the production of various kinds of heterologous proteins because of its high efficiency. At present, the recombinant expression in *P. pastoris* has mainly adopted an integrating mode for both single-promoter vectors and dual-plasmid vectors, and the methanol-induced promoter P*_AOX_* was always included [34,35,55]. However, the use of methanol in the large-scale production of proteins related to the food and healthcare industries has potential safety hazards due to its toxicity. The biggest advantage of constitutive promoters is that they do not require methanol for induction, and thus have a natural superiority over the AOX1 promoter for the food industry. To avoid the disadvantages caused by methanol induction, the constitutive P*_GAP_* and P*_GCW14_* promoters were used as independent initiation elements to construct a genome-integrative expression vector and an episomal expression vector in this study, and a novel dual-plasmid-based non-methanol induction *P. pastoris* expression system was constructed by combining these vectors.

During heterologous protein expression in recombinant *P. pastoris* strains containing a methanol-inducible promoter, the cells grow in the early period and the expression of recombinant proteins by induction takes place later. In contrast, protein expression in recombinant *P. pastoris* strains containing a constitutive promoter can take place along with the cell growth. Thus, an appropriate carbon source and its feed rate are key factors that affect the constitutive expression in large-scale, high-density fermentation processes. *P. pastoris* is capable of growing on a variety of carbon sources, including glucose, glycerol, oleic acid, and methanol. Methanol utilization is the poorest, glucose is mostly used for a shake flask culture, and glycerol is suitable for large-scale production [26]. A previous study in our laboratory also found that the expression of xylanase XynA using episomal vectors achieved the highest level when glycerol was used as the carbon source [54]. Results of this work proved that the most appropriate feed-flow rate of glycerol was 10% when *P. pastoris* was constitutively expressing recombinant heterologous proteins, indicating that both the appropriate carbon source and feed-flow rate are of vital importance for improving the expression efficiency. Furthermore, relative to methanol-induced expression, the constitutive expression had little effect on the physiological metabolism of the host cells and reduced the pressure on the host during the synthesis and secretion of heterologous proteins, thereby promoting the correct folding of the protein to maintain high specific activity. These properties will be very beneficial for expressing a variety of heterologous proteins with industrial usage on a large scale.

However, the protein yield under the constitutive expression for most enzymes was less than 5 g·L^−1^ in high-cell-density fermentation, which was much lower than that of methanol-induced expression. For instance, the constitutive expression level of β-mannanase from *Aspergillus niger* CBS 513.88 was 4.5 g·L^−1^ [56], and the expression level of endogenous β-glucanase AfCel12A from *A. fumigatus* was 3–5 g·L^−1^ [57]. However, the constitutive expression of α-amylase from barley in *P. pastoris* only reached 0.125 g·L^−1^ after high-cell-density fermentation [25]. With regard to xylanases, methanol-induction also has obvious superiority compared with traditional constitutive expression. The yield of *A. sulphureus*-derived xylanase reached 16.41 g·L^−1^ after a methanol-induced high-density fermentation [58], which was the highest value ever reported in a *P. pastoris* host. Another high yield case was 10.1 g·L^−1^ xynB from *Thermotoga maritima* that was obtained by methanol induction [59], while only 0.37 g·L^−1^ XylB from *A. niger* was yielded in *P. pastoris* X-33 after a large-scale fermentation using the constitutive pGAPZαA vector [60].

In comparison, the yield of XynA reached 13.6 g·L^−1^ using the novel dual-plasmid expression system constructed in this study, which was the highest constitutive expression level of xylanase reported so far to our knowledge, and was close to the highest reported methanol-induced yield of xylanase (16.41 g·L^−1^) [58]. It indicated that this novel, constitutive, dual-plasmid expression system has a strong expression ability. Further, the fermentation time of this strain was only 102 h, which is shortened by 30 h compared to the time cycle of methanol-induced, high-density fermentation. In addition, it was not necessary to add selective pressure during high-cell-density fermentation and strain preservation, which will significantly reduce the cost and improve the safety in the industrial fermentation of *P. pastoris*, since the number of reproductive generations in a fermentation cycle was less than 90, and the vectors had high genetic stability over this period. Moreover, since the metabolic pressure caused by glycerol culture on the yeast was significantly less than that caused by methanol induction, the synthesis of high specific activity protein in yeast was also improved. Cultivation using glycerol as the sole carbon source also eliminated the risks of methanol storage and methanol toxicity. All of these advantages give this novel constitutive dual-plasmid system more extensive application potentials in the large-scale production of industrial proteins, especially those that need more safety considerations. In a word, the *P. pastoris* expression system described here exhibited obvious advantages for the expression of high-value-added heterologous proteins with use in food or drugs, and could promote wider industrial applications of the *P. pastoris* expression system.

## Figures and Tables

**Table 1:** Xylanase activities of recombinant strains with a single promoter in shake flasks.

Strains	pGCW14-Episomal	pGCW14-Integrated	pGAP-Episomal	pGAP-Integrated
Activity (U·mL^−1^)	94.3 ± 7.6 ^a^	33.4 ± 2.1 ^b^	32.3 ± 2.5 ^b^	27.8 ± 1.9 ^c^

Note: Activity data was presented as mean ± standard deviation of triplicate determinations. The different letters indicate a statistically significant difference at *p* < 0.05 level for different strains.

**Table 2:** The genetic stability of the pGCW14-episomal plasmid in the host cell.

Generations	Number of Colonies		Gene Losing Rate
	Non-Selective	Resistance Selective	
30	143	140	2.1%
60	144	133	7.64%
90	170	144	15.29%

**Table 3:** Xylanase activities of recombinant strains with dual plasmids in shake flasks.

Strains	pGCW14-Integrated -pGAP-Episomal	pGAP-Integrated -pGCW14-Episomal	pGAP-Integrated -pGAP-Episomal	pGCW14-Integrated -pGCW14-Episomal
Activity (U·mL^−1^)	49.1 ± 3.5 ^a^	132.7 ± 9.7 ^b^	49.8 ± 3.7 ^a^	112.9 ± 7.2 ^c^

Note: Data was presented as mean ± standard deviation of triplicate determinations. The different letters indicate a statistically significant difference at *p* < 0.05 level for different strains.

**Table 4:** Comparison of XynA yields of the recombinant strain pGAP-integrated-pGCW14-episomal under different feed-flow rates of glycerol in a 3.6 L fermenter.

Yield	Feed Flow Rates of Glycerol			
	4%	6%	10%	12%
DCW (g·L^−1^)	162.68	191.1	239.12	241.08
Protein Conc. (g·L^−1^)	5.2 ± 0.37 ^a^	10.1 ± 0.95 ^b^	13.6 ± 1.1 ^c^	10.65 ± 0.87 ^b^
Enzyme activity (U·mL^−1^)	1720.3 ± 153.5 ^a^	2940.4 ± 210.1 ^b^	3925 ± 323.8 ^c^	3214 ± 276.9 ^b^
Specific activity (U·mg^−1^)	330.8 ± 25.2 ^a^	291.1 ± 22.3 ^b^	303.3 ± 28.1 ^ab^	301.7 ± 27.7 ^ab^

Note: Data was presented as mean ± standard deviation of triplicate determinations. The different letters indicate a statistically significant difference at *p* < 0.05 level for different data in the same row.

**Table 5:** Comparison of XynA yields for different recombinant strains in a 3.6 L fermenter.

Yield ^a^	DCW (g·L^−1^)	Protein Conc. (g·L^−1^)	Enzyme Activity (U·mL^−1^)	Specific Activity (U·mg^−1^)
KM71/pGAP-integrated ^b^	218.3	2.7	235	87
KM71/pGCW14-episomal	232.24	8.7 ± 0.52 ^a^	2512 ± 193.7 ^a^	298.1 ± 23.5 ^a^
KM71/pGCW14-episomal-pGAP-integrated	239.12	13.6 ± 1.1 ^b^	3925 ± 323.8 ^b^	303.3 ± 28.1 ^a^
KM71/pGCW14-integrated-pGAP-episomal	276.36	5.4 ± 0.37 ^c^	1056 ± 89.2 ^c^	195.6 ± 15.6 ^b^
KM71/pGAP-integrated-pGAP-episomal	251.86	5.08	968.3	190.6
KM71/pGCW14-integrated	237.65	4.86	847.6	174.4
KM71/pGCW14-episomal-pGCW14-integrated	243.04	11.56 ± 0.92 ^b^	3512 ± 290.3 ^b^	303.8 ± 22.9 ^a^

Note: Data was presented as mean ± standard deviation of triplicate determinations. The different letters indicate a statistically significant difference at *p* < 0.05 level for different strains in the same column. ^b^ Data comes from a previous work of our lab (Pan et al., 2018).

## Data Availability

Data is contained within the article or supplementary materials.

## Supplementary Materials

The following are available online at https://www.mdpi.com/article/10.3390/cimb43030161/s1.
