# Supplementary material for: Improved Production of Streptomyces sp. FA1 Xylanase in a Dual-Plasmid Pichia pastoris System
Source: Curr Issues Mol Biol. 2021 Dec 18;43(3):2289–304. doi: 10.3390/cimb43030161 (PMC8928940; doi:10.3390/cimb43030161)
Supplement: Supplementary file 1 [file cimb-43-00161-s001.zip › cimb-1507511-supplementary.pdf]

Supplementary Materials

Improved Production of *Streptomyces* sp. FA1 Xylanase in a Dual-Plasmid *Pichia pastoris* System

Wei Xia, Mengkai Hu, Yang Pan, Dan Wu and Jing Wu

Table S1. Primers used for plasmids construction.

Primer	Primer sequence (5′-3′)
gcw-F1	TTCGAAACGATGAGATTTCCTTC
gcw-R1	ATAGTTGTTCAATTGATTGAAATAGGGAC
gap-F1	CAATTGAACAACTATCAGGTGAACCCACCTAACTATT
gap-R1	TCTCATCGTTTCGAATTTGTTGTTTGAGTGAAGCGAG
gcw-F2	ATGTGAGCAAAAGGCCAGCA
gcw-R2	AGCTTGCAAATTAAAGCCTTCG
pars-F1	AAGGCTTTAATTTGCAAGCTTAGTGCTGATTATGATTTGACGTTTATAT
pars-R1	TGGCCTTTTGCTCACATG GCAACATCTTTGGATAATATCAGAATG
gap-R2	ATAGTTGTTCAATTGATTGAAATAGGGA
gcw-F3	CAATCAATTGAACAACTATCAGGTGAACCCACCTAACTAT
gcw-F4	AACTGCGTGCACTTCGTG
gcw-R3	GGTTTAGTTCCTCACCTTGTCG
kan-F1	ACAAGGTGAGGAACTAAACCATGAGCCATATTCAACGGGAA
kan-R1	CACGAAGTGCACGCAGTTTTAGAAAAACTCATCGAGCATCAAA

Table S2. The primers of qPCR.

Primers	Sequences(5′-3′)	reference
GAPDH-F	ACAAGGACTGGAGAGGTGGTAGAAC	(Sha et al., 2013)
GAPDH-R	GAGACAACGGCATCTTCAGTGTAAC	(Sha et al., 2013)
XynA-F	GCCCACTACAAGGGCAAGAT	this study
XynA-R	GACCTCGATCCAGTCGTTGC	this study

Table S3. qPCR analysis of xynA copy numbers of different recombinant strains.[a.]

KM71/pGAP-integrated				
Ct_{xynA}	Ct_{GADPH}	ΔCt	$2^{-\Delta Ct}$	Copy number
22.388	21.938	0.45	0.732042848	1.14±0.46
21.454	21.912	-0.458	1.373636233	
22.298	21.938	0.36	0.77916458	
21.174	21.912	-0.738	1.667862088	
KM71/pGAP-episomal				
Ct_{xynA}	Ct_{GADPH}	ΔCt	$2^{-\Delta Ct}$	Copy number
18.939	20.102	-1.163	2.239225777	2.05±0.17
19.032	19.898	-0.866	1.822602561	
19.543	20.598	-1.055	2.077718207	
19.675	20.718	-1.043	2.060507907	
KM71/pGCW14-episomal				
Ct_{xynA}	Ct_{GADPH}	ΔCt	$2^{-\Delta Ct}$	Copy number
18.845	19.84	-0.995	1.993080526	2.03±0.08
18.768	19.878	-1.11	2.158456473	
24.941	25.93	-0.989	1.984808749	
24.781	25.778	-0.997	1.995845438	
KM71/pGCW14-episomal-pGAP-integrated				
Ct_{xynA}	Ct_{GADPH}	ΔCt	$2^{-\Delta Ct}$	Copy number
18.431	19.919	-1.488	2.804998501	3.06±0.35
18.232	19.956	-1.724	3.30351066	
18.511	19.948	-1.437	2.707572558	
18.181	19.952	-1.771	3.412904392	

[a] Cycle threshold (Ct) values were shown, and quadruple parallel experiments were carried for each strain.

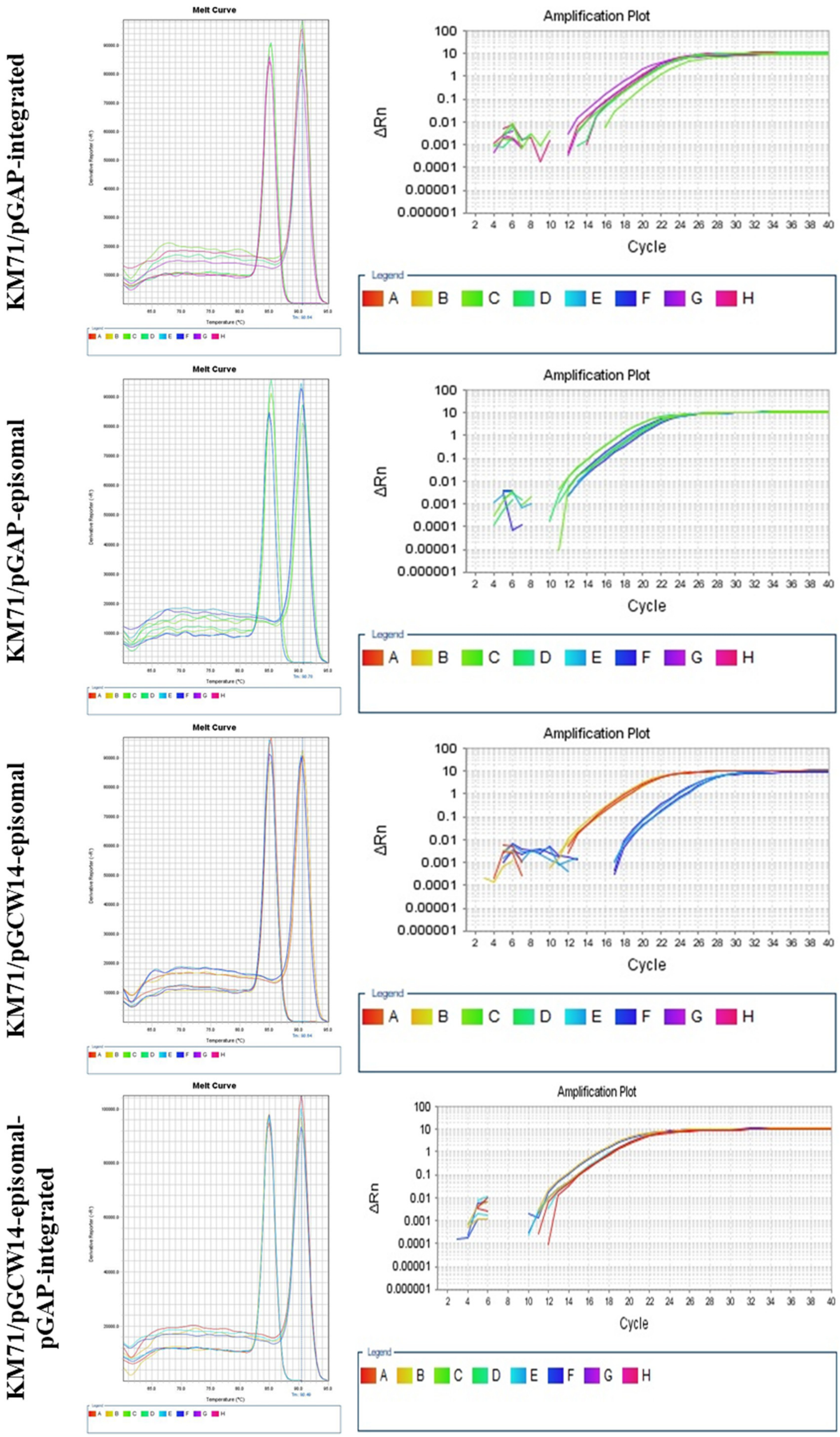

Figure S1. The melt curves and amplification plots of qPCR experiments for four expression strains.
